# Small and Intermediate Calcium-Activated Potassium Channel Openers Improve Rat Endothelial and Erectile Function

**DOI:** 10.3389/fphar.2017.00660

**Published:** 2017-09-20

**Authors:** Simon G. Comerma-Steffensen, Ingrid Carvacho, Elise R. Hedegaard, Ulf Simonsen

**Affiliations:** ^1^Department of Biomedicine, Pulmonary and Cardiovascular Pharmacology, Aarhus University Aarhus, Denmark; ^2^Animal Physiology, Department of Biomedical Sciences, Veterinary Sciences Faculty, Central University of Venezuela Maracay, Venezuela; ^3^Department of Biology and Chemistry, Faculty of Basic Sciences, Universidad Católica del Maule Talca, Chile

**Keywords:** erectile function, calcium activated potassium channels, NS309, NS4591, *corpus cavernosum*, K_Ca_2.3

## Abstract

Modulation of endothelial calcium-activated potassium (K_Ca_) channels has been proposed as an approach to restore endothelial function. The present study investigated whether novel openers of K_Ca_ channels with small (K_Ca_2.x) and intermediate (K_Ca_3.1) conductance, NS309 and NS4591, improve endothelium-dependent relaxation and erectile function. Rat *corpus cavernosum* (CC) strips were mounted for isometric tension recording and processed for immunoblotting. Mean arterial pressure (MAP), intracavernosal pressure (ICP), and electrocardiographic (ECG) measurements were conducted in anesthetized rats. Immunoblotting revealed the presence of K_Ca_2.3 and large K_Ca_ conductance (K_Ca_1.1) channels in the *corpus cavernosum*. NS309 and NS4591 increased current in CC endothelial cells in whole cell patch clamp experiments. Relaxation induced by NS309 (<1 μM) was inhibited by endothelial cell removal and high extracellular potassium. An inhibitor of nitric oxide (NO) synthase, and blockers of K_Ca_2.x and K_Ca_1.1 channels, apamin and iberiotoxin also inhibited NS309 relaxation. Incubation with NS309 (0.5 μM) markedly enhanced acetylcholine relaxation. Basal erectile function (ICP/MAP) increased during administration of NS309. Increases in ICP/MAP after cavernous nerve stimulation with NS309 were unchanged, whereas NS4591 significantly improved erectile function. Administration of NS309 and NS4591 caused small changes in the electrocardiogram, but neither arrhythmic events nor prolongation of the QTc interval were observed. The present study suggests that openers of K_Ca_2.x and K_Ca_3.1 channels improve endothelial and erectile function. The effects of NS309 and NS4591 on heart rate and ECG are small, but will require additional safety studies before evaluating whether activation of K_Ca_2.3 channels has a potential for treatment of erectile dysfunction.

## Introduction

Erectile dysfunction (ED) is defined as the persistent inability to attain and maintain an erection sufficient to permit satisfactory sexual performance (NIH Consensus Conference, 1993). ED is a highly prevalent, age-related global disease estimated to affect 300 million men in 2025 (McKinlay, 2000; Levy, 2002; Laumann et al., 2007; Wang et al., 2017). Phosphodiesterase type 5 (PDE5) inhibitors are recommended as first line treatment of ED due to their efficacy and safety profile (Hatzimouratidis et al., 2016). However, 20% of the general population of patients with ED and 50% of patients with diabetes and ED exhibit suboptimal responses to oral PDE5 inhibitors (Kendirci et al., 2006). Besides PDE5 inhibitors appears to have marginal effects when combined with other oral treatments (Dhir et al., 2011). Hence, this suggests that there is an unmet need for developing novel treatments for erectile dysfunction.

Activation of parasympathetic nerves to the penis leads to release of nitric oxide (NO), vasodilation of the penile arteries and relaxation of *corpus cavernosum* followed by blood filling of the penis and erection (Andersson, 2011). The rapid inflow of blood increases shear stress and leads to endothelium-dependent vasodilatation and further relaxation of *corpus cavernosum*. The calcium-activated potassium channels are involved both in the neurogenic phase as well as in the endothelium-dependent relaxations of erectile tissue. Thus, large-conductance calcium-activated K^+^ channels (K_Ca_1.1) were found to mediate the neurogenic and NO-induced relaxations in horse penile arteries (Simonsen et al., 1995), and the endothelium-dependent relaxations in rat penile arteries both in response to acetylcholine and to increases in flow (Kun et al., 2003; Schjørring et al., 2012). Moreover, openers of K_Ca_1.1 channels were found to relax erectile tissue (Spektor et al., 2002; Hewawasam et al., 2003; Kun et al., 2009) and penile arteries from man (Angulo et al., 2003a; Kun et al., 2008; Király et al., 2013). In mice lacking K_Ca_1.1 channels, erectile dysfunction was reported (Werner et al., 2005), and infusion of an opener of K_Ca_1.1 channels enhances the erectile responses in rats (Kun et al., 2009). In addition to K_Ca_1.1 channels, K_Ca_2.3 channels were suggested to be involved in endothelium-dependent vasodilatation in human and rat intracavernous penile arteries (Kun et al., 2008; Schjørring et al., 2012). In mesenteric small arteries from Zucker diabetic fatty rats relaxations induced by the endothelium-dependent vasodilator, acetylcholine can be restored by treatment with an opener of K_Ca_2.x and K_Ca_3.1 channels (Brøndum et al., 2010). Although K_Ca_2.3 and K_Ca_3.1 channels are involved in the enhancing effect of the drug calcium dobesilate on erectile function in diabetic rats (Angulo et al., 2003b), there have been no attempts to directly investigate whether modulation of the K_Ca_2.x and K_Ca_3.1 channels improves cavernous endothelial and erectile function.

NS309 (6,7-dichloro-1H-indole-2,3-dione 3-oxime) and NS4591 (4,5-dichloro-1,3-diethyl-1,3-dihydro-benzoimidazol-2-one) are potent openers of K_Ca_2.x and K_Ca_3.1 channels with K_Ca_2.x channels being 2-4 times less sensitive to these drugs than K_Ca_3.1 channels (Strøbæk et al., 2004; Hougaard et al., 2009). Whereas submicromolar concentrations of NS309 and NS4591 doubled K_Ca_2.3 and K_Ca_3.1 currents, the K_Ca_1.1 and voltage-gated K_V_7.4 (KCNQ4) channels were insensitive up to 10 μM of the compounds (Strøbæk et al., 2004; Hougaard et al., 2009), hence suggesting these drugs possess the potency and selectivity for exploring the role of K_Ca_2.x and K_Ca_3.1 channels in erectile function.

The present study investigated the expression of K_Ca_2.3 and K_Ca_3.1 channels in *corpus cavernosum*, and whether the openers of K_Ca_2.x and K_Ca_3.1 channels, NS309 and NS4591, improve endothelium-dependent relaxation in erectile tissue and erectile function in rats.

## Methods

### Animal welfare and ethical statements

The animal experiments were conducted in accordance with the regulations of the Ethics Committee of the Animal Care and Use Committee at Aarhus University (Denmark) and to the Danish Animal Law (2011/561-2011 and 2014-15-2934-01059), and also followed the ARRIVE (McGrath et al., 2010) and COPE guidelines. The animals were housed in the animal facility in Universal Euro III type long width cages with standard wood bedding and space for 2 rats with a clean environment, and a comfortable temperature (22–23°C) with 12:12 h light-dark cycles and they had free access to food and tap water.

### Animals and tissues

Male wistar rats of the *Hannover* strain, 250–400 g (12–16 weeks), were selected blindly, randonmized, then stunned, decapitated and exsanguinated, according to Danish regulations, in order to harvest *corpus cavernosum* (CC) and other tissues (heart, liver mesenteric arteries) in ice-cold physiological salt solution (PSS) for *in vitro* studies. For *in vivo* studies, the rats were fasted for 8 h before anesthesia with pentobarbital sodium (50 mg kg^−1^ i.p.). Adequate depth of anaesthesia was ensured by the absence of the toe pinch withdrawal reflex.

### Western blot

CC after myograph experiments or heart apex/liver/mesenteric arteries excised after rat sacrifice were snap frozen in liquid nitrogen and kept at −80°C until homogenization. Protein was extracted in lysis buffer (20 mmol L^−1^ tris HCL, 5 mmol L^−1^ EGTA, 150 mmol L^−1^ NaCl, 20 mmol L^−1^ glycorophosphate, 10 mmol L^−1^ NaF, 1% triton X-100, 0.1% tween-20, pH 7.5) using a Precellys 24 homogenizer (Bertin Technologies, Montigny-le-Bretonneux, France). The samples were exposed to 3 cycles at 5,000 rpm for 30 s each. After homogenization, they were left on ice for 20 min before being centrifuged for 15 min at 13,000 g at 4°C. The supernatant was frozen at −80°C. Total protein was quantified using the Bio-Rad Protein Assay (Bio-Rad, Hercules, CA, USA). Protein lysate was mixed with sample buffer and loaded onto the gel with a pre-stain marker (Bio-Rad, Hercules, CA, USA). The samples were loaded in different quantities (K_Ca_2.3 = heart: 0.5 μg, CC: 10 μg. K_Ca_3.1 = heart: 8 μg, CC: 12 μg. K_Ca_1.1 = CC, mesenteric arteries, liver and heart: 8 μg) and the proteins were separated by SDS-polyacrylamide gel electrophoresis (SDS-page) using a 4–12% Criterion XT Bis-Tris gel (Bio-Rad, Hercules, CA, USA) at 200 V in a criterion cell (Bio-Rad, Hercules, CA, USA). Transfer to a membrane was achieved for 1 h at 100 V in a criterion blotter (Bio-Rad, Hercules, CA, USA). The membranes were blocked in TBS-T with 0.3% I-block for 2-4 h before incubation with the primary antibodies in TBS-T with 0.3% I-block: K_Ca_2.3 (70 kDa, rabbit, 1:200), K_Ca_3.1 (46 kDa, rabbit, 1:200), K_Ca_ 1.1 alpha (~110 kDa, rabbit, 1:400), and pan actin (45 kDa, rabbit, 1:1,000). The specificity of the antibodies was checked. Thus, in previous studies we have observed the K_Ca_2.3 antibody gives a low expression in CC from down-regulated animals and high expression in K_Ca_2.3 overexpressing mice (Comerma-Steffensen et al., 2017). To test the K_Ca_3.1 antibody, samples from wildtype and K_Ca_3.1 mice were examined, and for the K_Ca_1.1 antibody CC samples were incubated in the absence and the presence of the peptide (APC-107, anti-K_Ca_1.1, Alomone) used for raising the antibody. All of the membranes were left overnight at 4°C. The membrane was then washed in TBS-T and incubated for 2 h in the secondary antibody goat anti-rabbit IgG conjugated to HRP (Santa-Cruz Biotechnology, Santa Cruz, CA, USA) (1:4,000). The membrane was developed using an ECL-Plus kit (GE Health care, Copenhagen, Denmark) and images sampled by a luminescence camera in an Image Quant LAS 4,000 mini from General Electrics. The marker was visualized by epi-luminicense. In each gel the same band width was used and band intensity was quantified by Image Quant TL software (Amersham Biosciences, Copenhagen, Denmark), and the results expressed as ratio to pan actin.

### Isometric tension recording in isolated *Corpus cavernosum*

After cutting the crura corpora cavernosa at the point of adhesion to the lower pubic bone, the penis was removed and submerged in ice-cold (4°C) PSS, and the CC was microsurgically dissected free. The CC strips were mounted between two clamps, one clamp grabbing a fixed wire and another attached to a wire connected to an isometric transducer in a 750TOBS myograph (DMT Technologies, Aarhus, Denmark). CC strips were immersed in 10 ml of PSS, bubbled with BioAir (5% CO_2_ and 19.82% O_2_ in nitrogen) and maintained at 37°C.

Four strips of CC from rats were mounted simultaneously. In case of comparing CC strips with and without endothelium, the endothelium was removed mechanically in two of the preparations as previously described (Kim et al., 1991). Thus, the strips were rubbed between the thumb and the index for 20 s. After rinsing in chilled PSS, the CC strips were gently rolled across a dry paper towel to generate shear forces across the endothelial surfaces of the lacunar spaces, and then mounted. In every case, basal tension was 1.3 ± 0.1 mN. The basal tension was reached in 30 min of stabilization and maintained afterwards for 45 min, while PSS was changed every 15 min to keep viability of the muscle (Hedlund et al., 1999; Kun et al., 2009). The viability of the preparation was examined by stimulation with KPSS (119 mM) and endothelial function examined by addition of acetylcholine (ACh) (1 μM) in preparations contracted with noradrenaline (NA) (1 μM). Endothelial cell removal was considered as successful only if relaxation was absent or changed to contraction in response to ACh.

### Concentration response curves

To investigate the role of potassium channels in acetylcholine relaxation, the strips were contracted with 1 μM NA or 1 μM NA on top of 60 mM KPSS before concentration-response curves were constructed for ACh, NS309, NS4591, and SNP.

To assess involvement of potassium channels, prostanoids and nitric oxide (NO), the preparations were incubated with either vehicle, or blockers of K_Ca_1.1 channels, iberiotoxin (10^−7^ M), of K_Ca_1.1 and K_Ca_3.1 channels, charybdotoxin (10^−7^ M), of K_Ca_3.1 channels, TRAM34 (10^−6^ M), of K_Ca_2.x channels, apamin (5 × 10^−7^ M), and of voltage-gated K_V_7 channels, linopirdine (10^−5^ M), inhibitors of cyclooxygenase, indomethacin (10^−5^ M) or of NO synthase (L-NOARG 10^−4^ M) for 30 min, then the preparations were contracted with NA (1 μM), and concentration-response curves for NS309 and/or ACh were constructed. To investigate whether opening of K_Ca_2.x and K_Ca_3.1 channels enhances acetylcholine relaxation, the preparations were incubated with NS309 (5 × 10^−7^ M) for 30 min, and concentration-response curves for acetylcholine were performed in NA-contracted CC strips.

### Measurement of intracavernous and mean arterial pressure (MAP)

The pentobarbital anaesthesized rats breathed spontaneously and body temperature was monitored continuously and maintained at 37°C by placing them on a heating blanket. A heparinized (100 IE mL^−1^) polyethylene (PE) catheter (PE 50) connected to a pressure transducer (Disposable BP Transducer, AD Instruments, Oxford, UK) was introduced into the carotid artery to measure MAP. Another pressure transducer connected to a 25-gauge needle attached to a heparinized catheter PE, was inserted into the crus CC, after scrotum incision, to measure the intracavernous pressure (ICP). Continuous direct measurements of MAP and ICP were obtained, registered and analyzed on a computerized data acquisition system (PowerLab, ADInstruments). A stabilizing period of 20–30 min was allowed before registration of basal ICP and MAP.

The cavernous nerve was isolated, after an incision in the abdominal middline, by locating the nerve running on the bladder and prostate area as previously described (Kun et al., 2009). The electrical stimulation was performed with a tip curve bipolar platinum electrode, which was connected to a S48 stimulator (Grass Instrument Co., Boston, MA, USA). NS309, NS4591, or vehicles (DMSO and PEG400) were administered by infusion in the jugular vein. Before the beginning of the experiment, three leads or electrodes, configured in an *Einthoven* configuration, were positioned to obtain the second lead derivation to access the electrical activity of the heart and the heart rate.

### Experimental protocol

A first stimulation of the cavernous nerve at parameters eliciting the maximum amplitude of the erectile response (square wave pulses of 6 Volts, 10 Hz, 1 ms for 30 s) was performed, as described earlier (Giuliano et al., 2003; Kun et al., 2009). Submaximal erectile response was attained, by changing the electrical parameters (0.6–1.55 Volts) until it was similar in magnitude to previous measurements (60–80% of maximal response). The rats were divided in four groups infused intravenously (200 μl) for 30 s with either NS309 (1 mg/kg), NS4591 (1 mg/kg), the vehicle for NS309 (DMSO), or the vehicle for NS4591, polyethylene glycol (PEG). The MAP and ICP was recorded during the infusion to observe whether there was a direct effect on erectile function, and then submaximal electrical stimulation responses were obtained at 3, 13, 23, and 33 min after the infusion to investigate whether the drugs facilitate the erectile responses. At the end of the experiment the maximal response was repeated to assure that the cavernous nerve was intact and the erectile function was normal. For each electrically induced erectile response, the ratio peak intracavernosal pressure (PICP) (mm Hg)/MAP (mm Hg) × 100 was measured, with PICP being the peak value reached by ICP during stimulation of the cavernous nerve.

### Electrocardiographic measurements during *in vivo* preparation

Recent studies have shown expression of K_Ca_2.3 channels in the atria and ventricle of the heart and proposed that blocking these channels can reverse atrial arrhythmia (Diness et al., 2011; Qi et al., 2014; Zhang et al., 2014; Torrente et al., 2017), and consequently we also examined the effect of NS309 and NS4591 on the electrical activity of the heart. ECG measurements were done for several minutes to assure normal rhythm from each animal before starting the experimental protocol. Signals were low-pass filtered by using the standard filter file for rat ECG recording. Data was obtained by the use of an animal bio-amplifier from AD Instruments. The software automatically obtained the different electrocardiographical values from AD Instruments. The rate-corrected QT interval (QTc) was calculated using the standard Bazett's formula (QTC = QT √RR^−1^).

### Primary isolation and culture of *Corpus cavernosum* endothelial cells

A fresh isolated CC strip was placed in sterile PBS and cut into small pieces together with collagenase II (400 mg ml^−1^, Worthington Biochemical Corporation, Lakewood, NJ, USA) for 60 min. at 37°C. The sampled tissue was centrifuged at 900 RPM with isolation of the pellet and transferred to a coated gelatin and collagen petri dish at 37°C. The culture media was Dulbecco's Modified Eagle Medium with Fungizone, penicillin streptomycin, new born fetal calf serum and HEPES. When the cells had reached confluence after 1–4 days, the cells were incubated with the endothelium specific CD102 [intracellular adhesion molecule 2 (ICAM2)] rat anti-mouse (BD Biosciences, Albertslund, Denmark) for 30 min followed by incubation with Dynabeads with sheep anti-rat IgG (Life Technologies, Oslo, Norway) for 30 min at 4°C. Trypsin was added to separate the cells from the petri dish and transferred to a dynamag 5TM (Life Technologies, Oslo, Norway) for a 2 min clean, the pellet was dissolved and left in a coated petri dish and further cultivation was performed in endothelial cell growth serum (Provitro AG, Berlin, Germany) until patch clamp experiments were performed.

### Electrophysiology

Endothelial cells from rat CC primary culture, between passages 1–5, were dissociated using trypsin 0.25% and seeded onto glass coverslips 1 h before the start of the experiments. Whole-cell currents using the patch-clamp technique were measured at 20–23°C using an HEKA 10 USB amplifier controlled by PatchMaster acquisition program. Pipettes of 3–5 MΩ resistance were made from glass capillaries (1B150F-4, WPI). Currents were recorded using an extracellular solution containing (in mM): 140 NaCl, 4 KCl, 10 Glucose, 10 HEPES, 2 CaCl_2_ and 1 MgCl_2_, pH: 7.3–7.4. The intracellular solution was prepared as described previously (Stankevicius et al., 2011) and contained (in mM): 30 KCl, 100 K-Aspartate, 1 MgCl_2_, 10 mM EGTA, 3 Na_2_ATP, 5 HEPES, and 8,5 CaCl_2_, pH: 7.2 Free [Ca^2+^] in the intracellular solution was estimated to be 1 μM. The osmolarity of all solutions was 290–320 mOsm. All voltages were corrected for calculated junction potentials present between the internal and external solution before seal formation. Potassium currents were activated by voltage steps from −140 to 140 mV in 40 mV increments for 250 ms, and using a holding membrane potential of −80 mV. Currents measured at a step of 100 mV were used to compare the effects of the openers or blockers used.

### Data and statistic analysis

The response to acetylcholine, NS309 or NS4591 are expressed as percentage relaxation of the contraction induced by NA (1 μM) or KPSS 60 mM plus 1 μM NA.

ECG data was assessed before starting every protocol, and before and after each electrical stimulation, also when any compound was administered to the rat. Statistical comparisons were performed by using Graphpad Prism 5.1 (GraphPad Software, San Diego, California, USA). Values were expressed as the arithmetic means ± S.E.M. Statistical comparisons between controls and treatments (TRAM-34, L-NOARG, apamin, charybdotoxin, iberiotoxin, NS309, indomethacin) or for western blot results, were performed by non-paired Student's *t*-test for two mean values, One-way ANOVA with a Tukey post-test or by two-way ANOVA with a Bonferroni post-test. *In vivo* results were assessed by one-way ANOVA with a Tukey post-test; otherwise a Dunnett's post-test was performed. Electrophysiological data were analyzed using IgorPro and Origin Pro and the values were expressed as means ± S.E.M. Statistical analyses were performed using Excel and included paired Student's *t*-test, two-tailed *P*-value and two-way ANOVA. Significance was considered at a minimal of *P* ≤ 0.05. Each group size was equal or higher than four animals, except the patch clamp experiments where three animals was the minimal sample, since viable endothelial cells where not obtained from additional three animals.

### Materials

Noradrenaline (NA), acetylcholine (ACh), N-omega-nitro-L-arginin (L-NOARG), sodium nitroprusside (SNP), Linopirdine and polyethylene-glycol (PEG) were purchased from Sigma–Aldrich (Saint Louis, MO, USA). Apamin, charybdotoxin, and iberiotoxin were acquired from Latoxan (Valence, France). 1-[(2-Chlorophenyl)diphenylmethyl]-1H-pyrazole (TRAM-34) was purchased from TOCRIS Bioscience (Abingdon, United Kingdom). NS309 and NS4591 were a donation by Neurosearch A/S (Ballerup, Denmark). Indomethacin was purchased at Bie and Berntsen A/S (Herlev, Denmark). PSS had the following composition (mM): CaCl_2_ 2.5, MgS0_4_-7H_2_O 1.17, NaHCO_3_ 25.0, NaCl 119, KH_2_P0_4_ 1.18, KCl 4.7, glucose 5.55, EDTA 0.026 (pH 7.4). The 119 or 60 mM K^+^ solution had the same composition as the PSS, but NaCl was replaced by KCl on an equimolar basis to reach the final 119 or 60 mM K^+^ concentration. All drugs were freshly prepared in stock solutions and kept at −20°C until the day of the experiment, or for the *in vivo* experiments made the day of the experiments. Drugs were dissolved in distilled water, PSS, DMSO, and PEG depending on the compound used.

K_Ca_2.3 antibody (SC28621) was from Santa Cruz Biotechnology (Santa Cruz, CA, USA), K_Ca_1.1 antibody (APC-151) and peptide (APC-107) were from Alomone (Jerusalem, Israel), K_Ca_3.1 antibody (CA1788) from Cellular Applications and pan-actin (4,968) from Cell Signaling Technology Inc. (Danvers, MA, USA). Secondary antibody was a polyclonal anti-rabbit from Invitrogen. The ECL-Plus kit was from GE Healthcare and I-block was from Applied Biosystems (CA, USA).

## Results

### K_Ca_ protein expression in *Corpus cavernosum*

**Figure 1:**
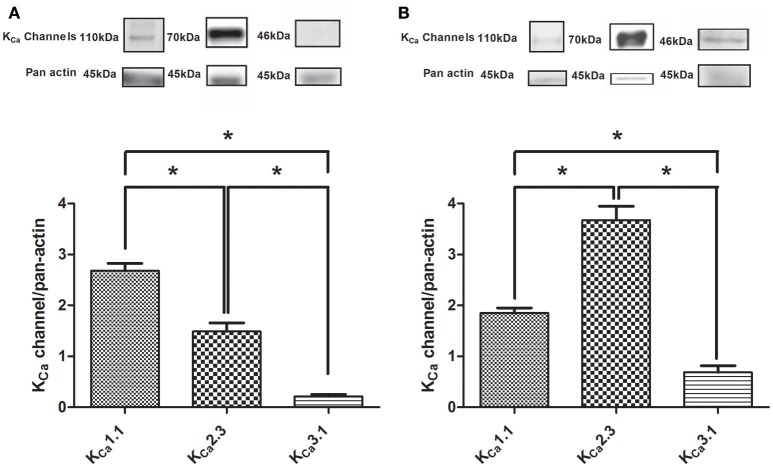
Immunoblotting of calcium-activated potassium channels in rat *corpus cavernosum* and the heart. **(A)** Immunoblotting of the K_Ca_1.1 alpha subunit (*n* = 11), K_Ca_2.3 (*n* = 14), and K_Ca_3.1 (*n* = 12) channels in *corpus cavernosum*. **(B)** Immunoblotting of the K_Ca_1.1 alpha subunit (*n* = 6), K_Ca_2.3 (*n* = 5), and K_Ca_3.1 (*n* = 3) channels in the heart. The results were expressed as a ratio to pan actin and show expression of mainly K_Ca_1.1 and K_Ca_2.3 immunoreaction in the *corpus cavernosum*. Data are expressed as means ± S.E.M. *P ≤ 0.05, one-way ANOVA followed by Student's *t*-test.

The expression of K_Ca_1.1 and K_Ca_2.3 channels as ratio to pan actin were abundant in rat CC and in the heart, while the expression of K_Ca_3.1 channels was markedly less (Figure 1). Both the expression of K_Ca_2.3 and K_Ca_3.1 were more pronounced in homogenates of rat heart (Figure 1B) compared to CC (Figure 1A), while this was not the case for the K_Ca_1.1 alpha subunit (Figure 1, Supplementary Figures S1–S3). Control experiments for K_Ca_3.1 showed there was no expression in heart samples from K_Ca_3.1 knockout mice (lane 1 Suppplementary Figure S3B), and in the presence of the anti-K_Ca_1.1 peptide, no immunoreactive bands were detected for K_Ca_1.1 in rat mesenteric arteries, CC, and liver (Supplementary Figure S2C).

**Figure 2:**
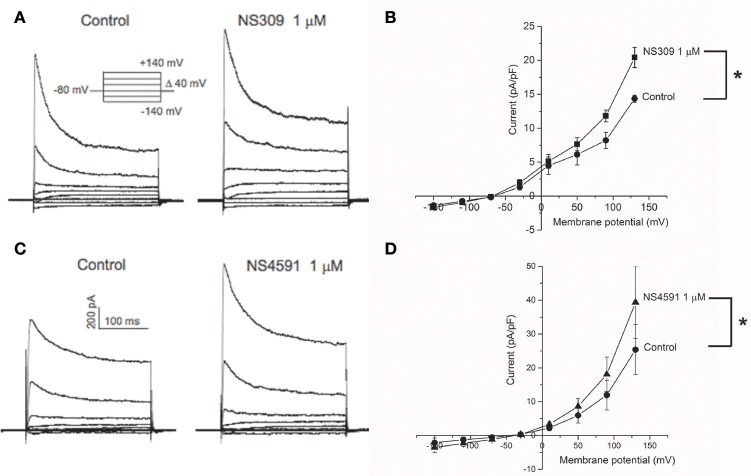
K_Ca_2.x channels are functionally expressed in endothelial cells from *corpus cavernosum*. Whole-cell voltage clamp recordings from endothelial cells derived from primary culture of *corpus cavernosum* **(A–D)**. **(A)** Current evoked from voltage steps from −140 to +140 in control cells (left) or in presence of NS309 (1 μM; right). **(B)** Current-Voltage (I-V) relation in basal conditions (filled circles) and in response to 1 μM NS309 (filled squares). Results are means ± SEM. *n* = 4. **(C)** Current evoked from voltage steps from −140 to +140 in control cells (left) or in presence of NS4591 (1 μM; right). **(D)** I-V relation for control cells (filled circles) and in response to 1 μM NS459 (filled triangles). Results are mean ± SEM, *n* = 4. *P ≤ 0.05.

To further assess the expression of K_Ca_2.x and K_Ca_3.1 channels in the endothelial cells from CC, we measured the activity of these channels using whole cell voltage clamp. Potassium currents were measured in response to a voltage step protocol (Figures 2A,C). The basal current was significantly blocked in the presence of apamin, while TRAM-34 failed to inhibit the current (Supplementary Figures S4A,B). The basal activities of the channels were potentiated by the opener NS309 (Figures 2A,B), and by NS4591 (Figures 2C,D), in agreement with the expression of K_Ca_2.x and K_Ca_3.1 channels (Strøbæk et al., 2004; Hougaard et al., 2009; Stankevicius et al., 2011).

To examine the calcium-dependency of the currents induced by NS309, HUVECs were investigated and showed NS309 increased current in the presence, but not in the absence of calcium as previously described (Supplementary Figure S5A), and these currents were reduced in the presence of apamin and abolished in the presence of TRAM-34 as previously described in HUVECs (Supplementary Figure S5B; Stankevicius et al., 2011).

### Functional studies in *Corpus cavernosum*

**Figure 3:**
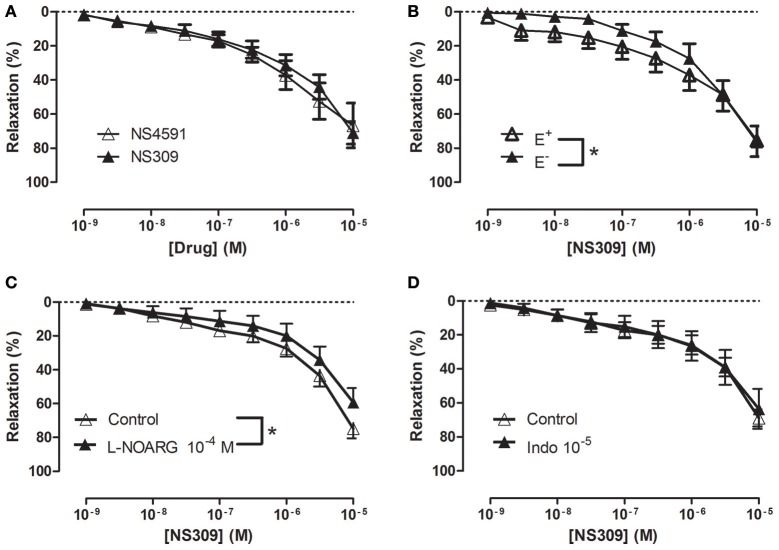
Involvement of the endothelium and nitric oxide (NO) in NS309 relaxation of rat *corpus cavernosum*. **(A)** Average concentration-response curves for NS309 (*n* = 10) compared with NS4591 (*n* = 11) in preparations with endothelium, **(B)** NS309 in preparations with endothelium (+E, *n* = 5) and without endothelium (−E, *n* = 5), **(C)** NS309 relaxation curves in the absence (*n* = 11) and the presence of nitro-L-arginine (L-NOARG 10^−4^ M, *n* = 7), and **(D)** NS309 relaxation curves in the absence (*n* = 6) and the presence of indomethacin (10^−5^ M, *n* = 6). Data are expressed as means ± S.E.M. *P ≤ 0.05, two-way ANOVA compared to preparations without endothelium or control preparations with endothelium.

**Figure 4:**
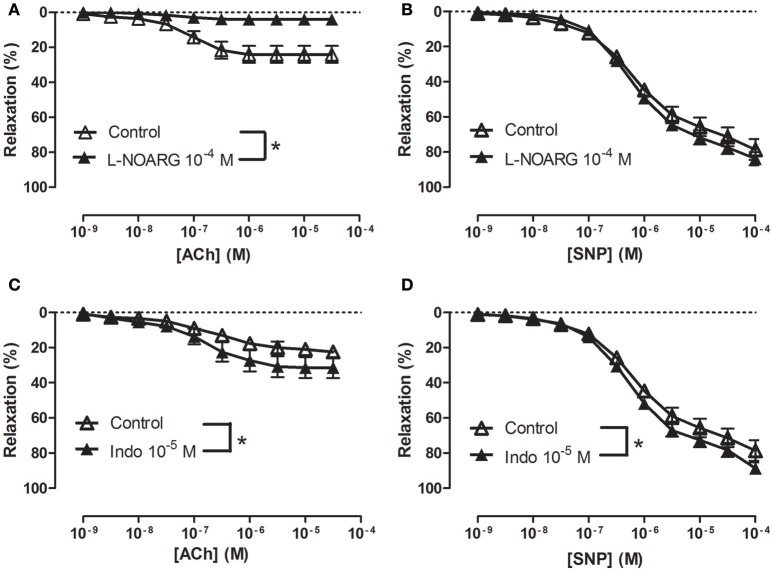
Involvement of nitric oxide (NO) in acetycholine (ACh) relaxation of rat *corpus cavernosum*. Concentration-response curves for **(A,C)** ACh (*n* = 7,10) and **(B,D)** sodium nitroprusside (SNP) (*n* = 7) in the absence and the presence of **(A,B)** an inhibitor of NO synthase, L-nitro-arginine (L-NOARG, 100 μM) (*n* = 10,7) or **(C,D)** cyclooxygenase, indomethacin (10 μM) (*n* = 7,7). Data are expressed as means ± S.E.M. *P ≤ 0.05, curves were significantly different, two-way ANOVA followed by a Bonferroni's post-test.

In NA (1 μM)-contracted CC strips with endothelium, the openers of K_Ca_2.x and K_Ca_3.1 channels, NS309 and NS4591 induced relaxations of comparable magnitude (Figure 3A, Supplementary Figure S6A). In preparations without endothelium, NS309 relaxation was reduced at concentrations <1 μM NS309, while relaxations to higher concentrations were similar in preparations with and without endothelium (Figure 3B). NS4591 relaxations were also reduced in preparations without endothelium (Supplementary Figure S6B). A NO synthase inhibitor, L-NOARG, significantly reduced NS309 (Figure 3C) and ACh relaxation, while SNP relaxation was unaltered (Figures 4A,B). Inhibition of cyclooxygenase with indomethacin, failed to inhibit relaxation to NS309 (Figure 3D), ACh, and SNP (Figures 4C,D).

**Figure 5:**
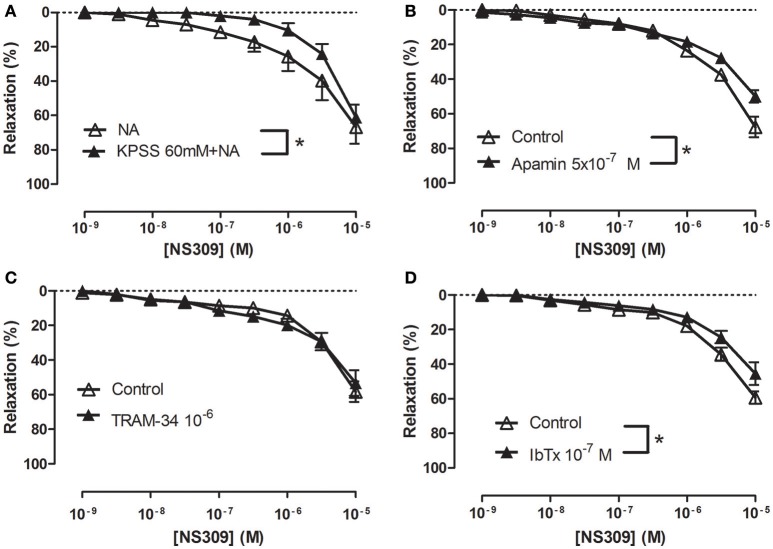
Role of potassium blockaged, apamin and iberiotoxin-sensitive channels in NS309 relaxations of rat *corpus cavernosum*. Average concentration-response curves for NS309 **(A)** in absence (*n* = 5) and the presence of KPSS 119 mM plus noradrenaline (*n* = 5), **(B)** in absence (*n* = 5) and the presence of a K_Ca_2 blocker apamin (0.5 μM, *n* = 5), **(C)** in the absence (*n* = 4) and the presence of a blocker of K_Ca_3.1 channels, TRAM-34 (1 μM, *n* = 4), **(D)** in the absence (*n* = 5) and the presence of a blocker of K_Ca_1.1, iberiotoxin (IbTx, 0.1 μM, *n* = 5). Data are expressed as means ± S.E.M. *P ≤ 0.05, two-way ANOVA compared to control preparations with endothelium.

To examine the effect of inhibition of potassium channels, the strips were contracted with 1 μM NA or with 60 mM KPSS plus 1 μM NA. The presence of 60 mM KPSS inhibited ACh-induced relaxation and significantly reduced relaxations induced by NS309 concentrations <1 μM (Figure 5A and Supplementary Figure S7A). The K_Ca_2.x blocker, apamin, and the K_Ca_1.1 blocker, iberotoxin, significantly reduced NS309 relaxation, while NS309 relaxation was unaltered in the presence of TRAM-34, a K_Ca_3.1 blocker or linopirdine, a blocker of K_V_7 channels (Figures 5B–D and Supplementary Figure S7B). These findings suggest that NS309 opens K_Ca_2 channels and that the endothelium-derived NO released by this activation leads to opening of smooth muscle K_Ca_1.1 channels, as previously described in rat mesenteric arteries (Stankevicius et al., 2011).

**Figure 6:**
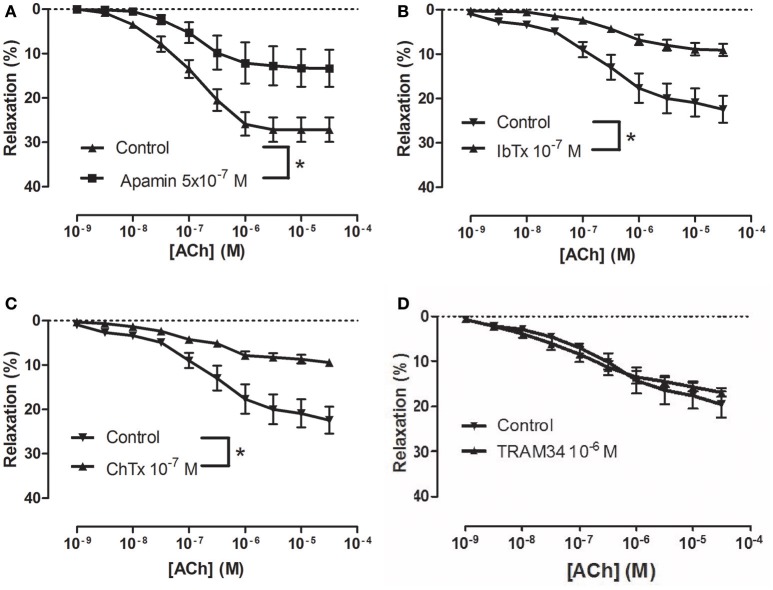
Involvement of apamin- and iberiotoxin-sensitive channels in acetylcholine (ACh) relaxation of rat *corpus cavernosum* (CC). Concentration-response curves for ACh in the absence and the presence of **(A)** control (*n* = 13) vs. apamin (0.5 μM) (*n* = 6), **(B)** control (*n* = 8) vs. iberiotoxin (0.1 μM) (*n* = 9), **(C)** control (*n* = 8) vs. charybdotoxin (0.1 μM) (*n* = 10), and **(D)** control (*n* = 8) vs. TRAM-34 (1 μM) (*n* = 9). Data are expressed as means ± S.E.M. *P ≤ 0.05, curves were significantly different, two-way ANOVA followed by a Bonferroni post-test.

**Figure 7:**
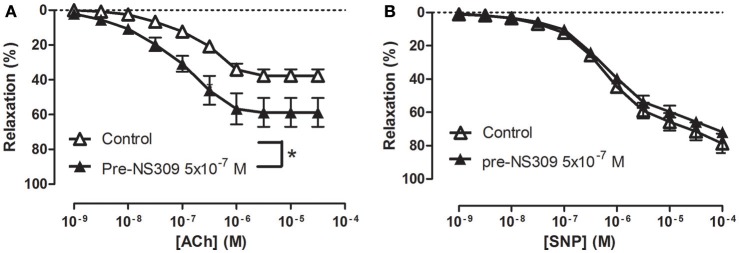
An opener of K_Ca_2.x and K_Ca_3.1 channels, NS309 selectively enhances acetylcholine relaxation in rat *corpus cavernosum*. Concentration-response curves for **(A)** acetylcholine (ACh) in the absence (*n* = 16) and the presence of NS309 (0.5 μM, *n* = 6), and **(B)** sodium nitroprusside (SNP) in the absence (*n* = 5) and in the presence of NS309 (*n* = 5) in noradrenaline (1 μM)-contracted *corpus cavernosum* strips. Data are expressed as means ± S.E.M. *P ≤ 0.05, curves were significantly different, two-way ANOVA followed by a Bonferroni's post-test.

To examine how NS309 may modulate ACh relaxation, the potassium channels involved in ACh relaxation were examined. ACh-induced relaxation was significantly reduced in the presence of apamin, a K_Ca_2.x blocker (Figure 6A), and iberiotoxin, a blocker of K_Ca_1.1 channels (Figure 6B), and charybdotoxin, a blocker of both K_Ca_3.1 and K_Ca_1.1 channels (Figure 6C), whereas ACh-induced relaxation was not affected by TRAM-34, a K_Ca_3.1 blocker (Figure 6D). These results suggest that the ACh relaxation is mediated by K_Ca_2.x and K_Ca_1.1 but not by K_Ca_3.1. Incubation with NS309 (5 × 10^−7^ M) markedly enhanced ACh relaxation, while SNP relaxations were unchanged (Figures 7A,B).

### Effect of NS309 and NS4591 on mean arterial and intracavernous pressure

**Figure 8:**
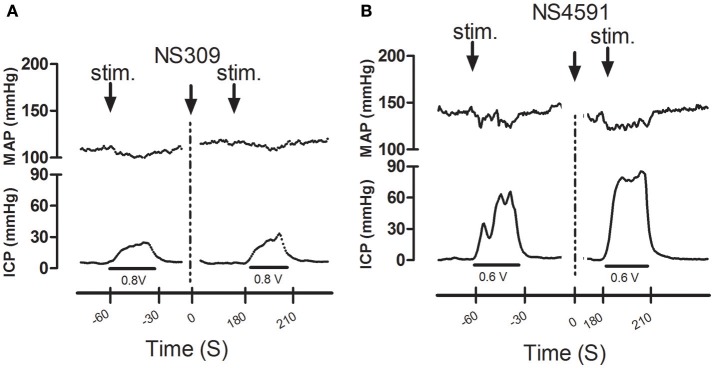
Original recordings showing the effect of NS309 and NS4591 on mean arterial pressure (MAP) intracavernous pressure (ICP) in rat penis. The upper traces show mean arterial pressure (MAP) and the lower traces the changes in intracavernous pressure (ICP) induced by submaximal stimulation of the cavernous nerve before and 3 min after **(A)** NS309 and **(B)** NS4591 in the rat.

**Figure 9:**
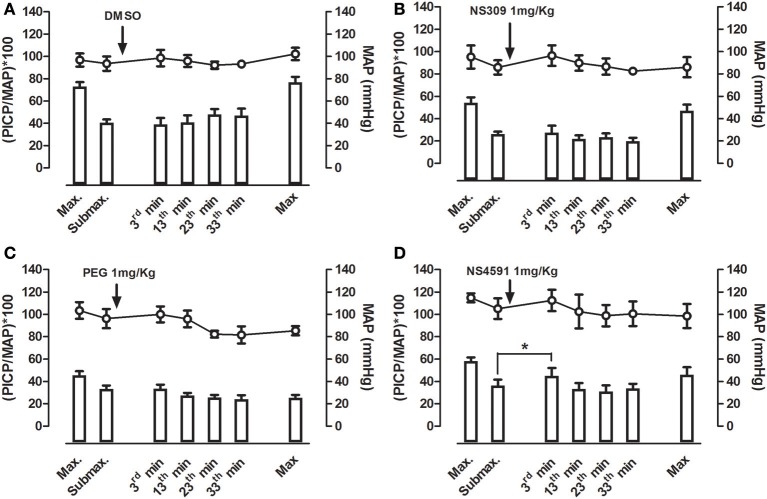
Average effect of NS309 and NS4591 on mean arterial pressure (MAP) and erectile function measured as peak intracavernosal pressure (PICP) over MAP in rats. A maximal and submaximal response to electrical stimulation was obtained before infusion of either vehicles, **(A)** dimethylsulpoxide (DMSO, 1 mgKg^−1^, *n* = 7), **(B)** polyethylene glycol (PEG, 1 mgKg^−1^, *n* = 7), **(C)** NS309 (1 mgKg^−1^, *n* = 5), or **(D)** of NS4591 (1 mgkg^−1^, *n* = 6), and the submaximal stimulations were performed 3, 13, 23, and 33 min after drug infusion. Finally, a maximal stimulation of the cavernous nerve was performed. The results show a facilitating effect on submaximal stimulation 3 min after NS4591 infusion. The direct effects of the drugs on MAP and intracavernosal pressure are depicted in Supplementary Figure S7. Data are expressed as means ± S.E.M. *P ≤ 0.05 compared with submaximal control response in the same animals using one-way ANOVA followed by Dunnett's test.

Infusion of NS309 caused a small increase in erectile function (ICP/MAP) and decrease in MAP, while NS4591 significantly decreased MAP without changing ICP/MAP compared to vehicle suggesting that there is no direct effect on the basal erectile state of these drugs (Supplementary Figure S8). To examine whether these drugs facilitate erectile function, submaximal electrical stimulation of the cavernous nerve was performed before and after infusion of NS309 and NS4591 (Figure 8), and the original recordings show a marked increase in intracavernosal pressure 3 min after infusion of NS4591 (Figure 8B). Due to the depth of anesthesia and different responses in the animals, the maximal and submaximal responses to electrical stimulation of the cavernous nerve may vary, and therefore the effect of the drugs were compared within each animal (Figure 9; Kun et al., 2009), but the submaximal erectile response was in the range of 20–30 mmHg (Figures 9A–D), while the MAP remained in the range 100–120 mmHg during the stimulation protocols (Figures 9A–D). Infusion of DMSO and NS309 failed to change the response to submaximal stimulation of the cavernous nerve 3 min after infusion (Figures 9A–C). In response to infusion of the vehicle PEG (1 mg/kg) and NS4591, erectile function (PICP/MAP^*^100) was further increased, respectively, 0.2 ± 2.0 (*n* = 7) and 8.7 ± 3.0 (*P* < 0.05, Student *t*-test, *n* = 6) in response to submaximal stimulation of the cavernous nerve 3 min after infusion (Figures 8B, 9D), suggesting a facilitating effect on erectile function. However, the effect of NS4591 was not maintained at 13, 23, or 33 min after the administration of the compound (Figure 9D).

### Effect of NS309 and NS4591 on the electrical activity of the heart

**Figure 10:**
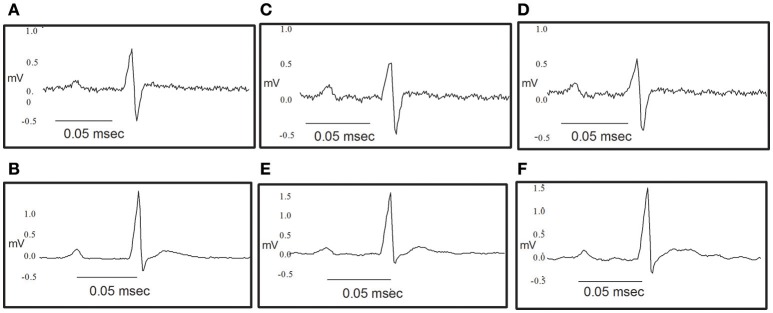
Original traces showing the effect of NS309 and NS4591 on the electrocardiography of the rat heart. **(A,B)** Representative ECG obtained as a control value for NS309 and NS4591. **(C)** Representative ECG obtained during NS309 administration. **(D)** Representative ECG obtained after NS309 administration. **(E)** Representative ECG obtained during NS4591 administration. **(F)** Representative ECG obtained after NS4591 administration.

The heart rate was reduced during the administration of NS309, progressing to an increment at the end of the administration, but this was not the case for administration of NS4591, where lower heart rate was observed (Tables 1A, 2A). There were no other changes in heart rate for NS309. The lead II of the ECG shows that during administration of NS309 and NS4591, the P wave amplitude was significantly increased after administration of NS309, but not during NS4591 infusion (Figure 10, Tables 1B, 2B). The PR interval shortened, during and after administration of NS309 and NS4591. The QTc interval was reduced during administration of NS309 and NS4591 (Tables 1B, 2B) but it was recovered afterwards for NS309. However, in the case of NS4591 the recovery took longer than 10 min. Ventricle repolarization was shorter between the 3rd and 10th minutes after NS309 administration, and there was no prolongation of the QTc interval (Tables 1B, 2B).

**Table 1:** **(A)** NS309 erectile function measurements and **(B)** electrocardiography during the experimentation protocol.

	**Control**	**Before adm. NS309**	**During adm. NS309**	**After adm. NS309**	**3 min**	**10 min**	**20 min**	**30 min**
**A**								
HR (beats)	362 ± 2.1^A^	344 ± 1.1^B1^	338 ± 1.3^B1^	368 ± 1.3^2^	362 ± 1.2	351 ± 0.7	358 ± 1.5	349 ± 1.4
MAP (mmHg)	85.9 ± 6.4	89.5 ± 8.7	79.5 ± 10.3	95.3 ± 7.1	96.3 ± 9.2	89.8 ± 6.9	86.6 ± 7.3	82.5 ± 1.8
ICP (mmHg)	22.5 ± 1.5^A^	6.2 ± 0.7^B^	7.2 ± 0.7^B^	5.7 ± 0.5^B^	24.3 ± 4.9	19.4 ± 3.3	20.5 ± 4.1	16.1 ± 2.2
ICP/MAP*100	26.3 ± 2^A^	6.6 ± 0.7^B1^	8.8 ± 0.7^B1^	5.9 ± 0.6^B2^	27.5 ± 6.2	22 ± 3.2	23.4 ± 3.4	20 ± 2.9
**B**								
P wave duration (ms)	0.0175 ± 0.0004	0.0165 ± 0.0004	0.0163 ± 0.0006	0.0158 ± 0.0002	0.01605 ± 0.0002	0.01669 ± 0.0004	0.01547 ± 0.0002	0.0164 ± 0.0005
P wave amplitude (mV)	0.1093 ± 0.0013^A^	0.06 ± 0.0031^B1^	0.0864 ± 0.0059^C2^	0.1166 ± 0.0016^3^	0.1178 ± 0.0013	0.1095 ± 0.0015	0.1148 ± 0.0016	0.0895 ± 0.0037^C^
PR interval (ms)	0.0491 ± 0.0002^A^	0.0472 ± 0.0003^B1^	0.0461 ± 0.0006^B1^	0.0441 ± 0.0004^C2^	0.0484 ± 0.0002	0.0483 ± 0.0004	0.0472 ± 0.0002^B^	0.0472 ± 0.0004^B^
PR segment (ms)	0.0320 ± 0.0004	0.0306 ± 0.0003^B^	0.0303 ± 0.0004	0.0320 ± 0.0002	0.0320 ± 0.0002	0.0315 ± 0.0002	0.0318 ± 0.0002	0.0312 ± 0.0002
QRS interval (ms)	0.0138 ± 0.0003^A^	0.0184 ± 0.0005^B1^	0.0160 ± 0.0002^C2^	0.0141 ± 0.0003^D3^	0.0144 ± 0.0003	0.0153 ± 0.0001	0.0144 ± 0.0002	0.0144 ± 0.0003
QTc (ms^1/2^)	0.1431 ± 0.0025^A^	0.1354 ± 0.0022^1^	0.1234 ± 0.0038^B2^	0.15 ± 0.0026^3^	0.158 ± 0.0028	0.1457 ± 0.0041	0.1568 ± 0.0037	0.1478 ± 0.0046
Q amplitude (mV)	0.0020 ± 0.0014^A^	−0.0155 ± 0.0010^B^	−0.0154 ± 0.0024^B^	−0.0104 ± 0.0027^B^	−0.0148 ± 0.0032^C^	−0.0042 ± 0.0010	−0.0006 ± 0.0016	0.0002 ± 0.0016
R amplitude (mV)	0.4566 ± 0.0104^A^	0.5266 ± 0.0074^B1^	0.4737 ± 0.0043^B2^	0.3996 ± 0.0111^C3^	0.4439 ± 0.0080	0.4779 ± 0.0049	0.4484 ± 0.0083	0.438 ± 0.0088
S amplitude (mV)	−0.4209 ± 0.0120^A^	−0.3564 ± 0.0062^B1^	−0.4102 ± 0.0119^2^	−0.4808 ± 0.0144^C3^	−0.4839 ± 0.0096^C^	−0.4535 ± 0.0109	−0.4639 ± 0.0122	−0.4524 ± 0.0116
ST height (ms)	0.05717 ± 0.0001^A^	0.0599 ± 0.0008^1^	0.0550 ± 0.0018^2^	0.0634 ± 0.0011^B1^	0.0484 ± 0.0011^C^	0.0482 ± 0.0007^C^	0.0536 ± 0.0010	0.0561 ± 0.0011
T amplitude	0.0713 ± 0.0010^A^	0.0707 ± 0.0007	0.0735 ± 0.0018	0.0710 ± 0.0009	0.0634 ± 0.0010^B^	0.0645 ± 0.0009^C^	0.0691 ± 0.0009	0.0696 ± 0.0010

*Significance (P ≤ 0.05) control submaximal stimulation vs. other submaximal stimulations (different letters). Significance (P ≤ 0.05) before, during and after NS309 administration (different numbers). One way ANOVA with a Tukey's or Dunnet's post-test*.

**Table 2:** **(A)** NS4591 erectile function measurements and **(B)** electrocardiography during the experimentation protocol.

	**Control**	**Before adm. NS4591**	**During adm. NS4591**	**After adm. NS4591**	**3 min**	**10 min**	**20 min**	**30 min**
**A**								
HR (beats)	395 ± 1^A^	420 ± 3^B1^	384 ± 6^A,D2^	409 ± 3^C,D1^	390 ± 3	370 ± 1.9^D^	362 ± 1.6^E^	366 ± 1.4^E^
MAP (mmHg)	105.1 ± 9.2	114.9 ± 8.6^1^	75.9 ± 7.1^2^	133 ± 4.9^1^	112.4 ± 9.6	102.5 ± 15.1	98.9 ± 9.6	100.5 ± 11
ICP (mmHg)	38.7 ± 7.3^A^	7 ± 0.95^C^	7.9 ± 0.95^C^	7.4 ± 0.96^C^	50.6 ± 9.3^A^	34.4 ± 7.2^A^	31 ± 6^A^	32.5 ± 5.4^A^
ICP/MAP*100	36.4 ± 5.2^A^	6.6 ± 1.4^B1^	10.8 ± 1.7^B1^	5.6 ± 0.7^B2^	45.1 ± 7^C^	33.4 ± 5.2	31.2 ± 5.3	33.9 ± 4.1
**B**								
P wave duration (ms)	0.0143 ± 0.0003	0.0138 ± 0.0002	0.0147 ± 0.0007	0.0123 ± 0.0005	0.0147 ± 0.0007	0.0128 ± 0.0006	0.0151 ± 0.0006	0.0134 ± 0.0002
P wave amplitude (mV)	0.1555 ± 0.0083^A^	0.1763 ± 0.0048^C1^	0.1060 ± 0.0029^D2^	0.1074 ± 0.0039^D2^	0.1186 ± 0.0026^D^	0.1070 ± 0.0064^D^	0.1399 ± 0.0021	0.1339 ± 0.0034^C^
PR interval (ms)	0.0465 ± 0.0004^A^	0.0457 ± 0.0001^A1^	0.0432 ± 0.0007^B1^	0.0433 ± 0.0004^B2^	0.0428 ± 0.0005^B^	0.0428 ± 0.0003^B^	0.0453 ± 0.0005	0.0430 ± 0.0005^B^
PR segment (ms)	0.0322 ± 0.0003^A^	0.0319 ± 0.0002^1^	0.0291 ± 0.0006^2^	0.0309 ± 0.0004^3^	0.0282 ± 0.0009^B^	0.0302 ± 0.0007	0.0307 ± 0.0006	0.0292 ± 0.0004^C^
QRS interval (ms)	0.0149 ± 0.0001^A^	0.0146 ± 0.0001^1^	0.0152 ± 0.0005^1^	0.0153 ± 0.0002^2^	0.0141 ± 0.0003	0.0161 ± 0.0002^B^	0.0157 ± 0.0001	0.0157 ± 0.0001
QTc (ms^1/2^)	0.1507 ± 0.0036^A^	0.1507 ± 0.0016^A1^	0.1235 ± 0.0023^B2^	0.1360 ± 0.0029^C3^	0.1325 ± 0.0043^C^	0.1230 ± 0.0138^B^	0.1446 ± 0.0019	0.1375 ± 0.0024
Q amplitude (mV)	0.0077 ± 0.0018	−0.0133 ± 0.0015^1^	−0.0003 ± 0.0016^2^	−0.0017 ± 0.0016^2^	−0.0007 ± 0.0018	−0.0015 ± 0.0015	−0.0048 ± 0.0023	0.0056 ± 0.0015
R amplitude (mV)	0.9819 ± 0.0160^A^	1.017 ± 0.0138^A1^	0.8065 ± 0.0501^B2^	0.7553 ± 0.0259^B2^	0.7335 ± 0.0274^B^	0.7810 ± 0.0285^B^	0.9913 ± 0.0111^A^	0.8419 ± 0.0302^B^
S amplitude (mV)	−0.2001 ± 0.0193^A^	−0.2391 ± 0.0109^A1^	−0.0768 ± 0.0220^B2^	−0.1435 ± 0.0068^C3^	−0.0616 ± 0.0132^B^	−0.1727 ± 0.0081	−0.1735 ± 0.0117	−0.1406 ± 0.0124^C^
ST height (ms)	0.1127 ± 0.0027^A^	0.1202 ± 0.0057^1A^	0.0860 ± 0.0051^2A^	0.0645 ± 0.0055^B2^	0.0779 ± 0.0040^B^	0.0723 ± 0.0097^B^	0.1285 ± 0.0046	0.0903 ± 0.0065
T amplitude	0.1996 ± 0.0040^A^	0.2239 ± 0.0070^1^	0.1579 ± 0.0115^2^	0.1506 ± 0.0086^B2^	0.1500 ± 0.0080^B^	0.1581 ± 0.0127^C^	0.2268 ± 0.0053	0.1784 ± 0.0111

*Significance (P ≤ 0.05) control submaximal stimulation vs. other submaximal stimulations (different letters). Significance (P ≤ 0.05) before, during and after NS4591 administration (different numbers). One way ANOVA with a Tukey's/Dunnet's or Student's post-test*.

## Discussion

The main findings of the present study are the findings of K_Ca_1.1 and K_Ca_2.3 channels expression, and that apamin- and iberiotoxin-sensitive channels are involved in ACh-relaxation in rat CC. Moreover, openers of K_Ca_2.x and K_Ca_3.1 channels, NS4591 and NS309, respectively, for first time has been shown to improve erectile function and enhance endothelium-dependent relaxations, while the effects on heart rate and electrical activity appear relatively small.

### Expression of calcium-activated potassium channels

The calcium-activated K^+^ channels have been reported to be expressed in the erectile tissue. Using RT-PCR and Northern blotting suggested the presence of K_Ca_2.3 and K_Ca_3.1 in human (Chen et al., 2004) and rat *CC* (Zhu et al., 2010). Immunohistochemical studies further revealed the expression of K_Ca_2.3 and K_Ca_3.1 in the endothelium of rat and human penile arteries, while the authors suggested mainly K_Ca_1.1 channels are expressed in *CC* smooth muscle (González-Corrochano et al., 2013). Moreover, our recent electron microscopic studies of K_Ca_2.3 channels showed immunoreactivity for these channels in the apical-lateral membrane of endothelial cells in the mouse *CC* (Comerma-Steffensen et al., 2017), and our qPCR studies showed abundant expression of K_Ca_1.1 and K_Ca_2.3 channels in mouse *CC* (Comerma-Steffensen et al., 2017). The present immunoblotting studies showed the expression of mainly K_Ca_1.1 and K_Ca_2.3 channels in *CC*, while the expression of K_Ca_3.1 channels appeared to be sparse. Thus, the present findings support that mainly K_Ca_1.1 and K_Ca_2.3 channels are expressed in rat *CC*.

Calcium-activated potassium channels are expressed in the heart; although their function remains controversial (Nagy et al., 2009; Torrente et al., 2017). K_Ca_2.1-2.3 were found to be expressed in both mouse and human atrias-ventricles (Xu et al., 2003; Tuteja et al., 2005). The K_Ca_3.1 channels are also expressed in the mouse heart, and they are upregulated during cardiac fibrosis (Ju et al., 2015). The present study provides further support to previous findings confirming the expression of K_Ca_2.3, and K_Ca_3.1 channels in the heart.

### Involvement of calcium-activated potassium channels in relaxation of erectile tissue

In addition to K_Ca_1.1 channels, K_Ca_2.3 channels were suggested to be involved in endothelium-dependent vasodilatation in human and rat intracavernous penile arteries (Kun et al., 2008; Schjørring et al., 2012; Király et al., 2013). Here, the endothelium-dependent vasodilator, acetylcholine, relaxed *CC* by a mechanism sensitive to inhibition of NO synthase, high extracellular potassium, and also to inhibition by apamin and iberiotoxin, suggesting an involvement of K_Ca_1.1 and K_Ca_2.x channels in the relaxations of rat *CC*. Charybdotoxin, which is considered a blocker of K_Ca_3.1 and K_Ca_1.1 channels, inhibited acetylcholine relaxation as well, while a selective blocker of K_Ca_3.1 channels, TRAM-34 failed to change the relaxations induced by acetylcholine. All together our results suggest that K_Ca_1.1 and K_Ca_2.x channels are involved in acetylcholine relaxation of rat CC.

Openers of K_Ca_2.x and K_Ca_3.1 channels have been found to enhance endothelium-dependent vasodilatation. In retinal arteries, NS309 was found to enhance NO-mediated relaxations induced by the endothelium-dependent vasodilator, bradykinin (Dalsgaard et al., 2010) and NS309 also enhanced EDH-type relaxations induced by acetylcholine in mesenteric arteries from control rats, and restored EDH relaxations in arteries from diabetic rats (Brøndum et al., 2010). In the present study, NS309 by itself induced concentration-dependent relaxations, which at low concentrations (nM) were blocked by high extracellular potassium ^+^NA and by apamin/iberiotoxin suggesting involvement of K_Ca_2.x or K_Ca_1.1 channels in these relaxations. Moreover, this is the first time low concentrations of NS309 have been shown in the rat CC to enhance the concentration-response curves for acetylcholine. The effect appeared to be specific given that NS309 failed to change concentration-response curves for the NO donor, SNP. Therefore, NS309 at low concentrations induces marked potentiation of acetylcholine relaxation of *CC in vitro*.

NS309 and NS4591 are both considered openers of K_Ca_2.x and K_Ca_3.1 channels. In whole-cell patch-clamp experiments, 45 and 530 nM of NS4591 were required for doubling of, respectively, K_Ca_3.1 and K_Ca_2.3 current (Hougaard et al., 2009), while 10 nM and 30 nM NS309 were required for doubling, respectively, K_Ca_3.1 and K_Ca_2.3 currents in HEK293 cells overexpressing these channels (Strøbæk et al., 2004). In HUVECs NS309 (0.1 μM) induced calcium-dependent currents which were inhibited in the presence of apamin and TRAM-34, while iberotoxin failed to change the responses to NS309 (Supplementary Figure S5; Stankevicius et al., 2011). In whole cell voltage clamp experiments in the endothelial cells from *CC*, it appears from the reversal potentials that other channels, probably cationic non-selective channels from the Transient Receptor Potential (TRP) channels, also are expressed in *CC* endothelial cells shifting the reversal potential by cationic transport through the membrane toward depolarized membrane voltages (Figure 2). TRP channels have been reported to be expressed in endothelial cells (Nilius and Droogmans, 2001; Wandall-Frostholm et al., 2015), and further studies should address which particular TRP channels is/are expressed in primary culture of rat *CC* endothelial cells. However, whole cell voltage clamp experiments in the endothelial cells from *CC*, showed increased K^+^ currents in response to both openers (1 μM). Taken together, these findings support that NS309 and NS4591 at low concentrations leads to opening of calcium-dependent K^+^ channels in *CC* endothelial cells.

The low concentrations of NS309 (<1 μM) leading to opening of apamin and TRAM-34-sensitive K^+^ channels in whole cell patch clamp experiments of HUVECs (Supplementary Figure S5; Stankevicius et al., 2011) and increase of current in *CC* endothelial cells, induced only small relaxations in *CC* of the rat which were insensitive to inhibition of TRAM-34. The low expression of K_Ca_3.1 channels in rat *CC* may explain the lack of sensitivity to TRAM-34 (Figure 1). In the intact rat *CC* contracted by NA, the endothelium-derived factors, e.g., NO released by activation of the endothelial K^+^ channels will have to repolarize and/or decrease smooth muscle cell calcium and therefore, higher concentrations of the K^+^ channel opener may be required. Moreover, in the patch clamp studies of the HUVECs, the intracellular calcium concentration in the pipette was 1 μM, and likely the calcium concentration is lower in the endothelial cell layer of the intact *CC*, and that may explain that the modest relaxations induced by low concentrations of NS309 and NS4591. The latter explanation is also indirectly supported by the observation that in the presence of ACh, previously found to cause marked increases in endothelial cell calcium of intact rat mesenteric arteries (Stankevicius et al., 2006; Brøndum et al., 2010), a low concentration of NS309 (0.5 μM) induced a marked leftward shift in the concentration-response curve for ACh.

We have previously reported that an opener of K_Ca_1.1 channels, NS11021 failed to increase current in HUVECs (Kun et al., 2009), and iberiotoxin failed to change currents induced by NS309 in HUVECs (Supplementary Figure S5). However, iberiotoxin inhibited NS309 relaxations in CC strips. Although K_Ca_1.1 channel expression and/or iberiotoxin-sensitive channels have been described both in the endothelium of renal arteries (Brakemeier et al., 2003) and to be involved in NO-induced relaxation of the smooth muscle of the penile artery and erectile smooth muscle (Simonsen et al., 1995; Kun et al., 2009), our findings suggest that iberiotoxin-sensitive channels are involved in ACh and NS309 relaxations of rat CC, likely by a mechanism involving NO-induced activation of K_Ca_1.1 channels in the smooth muscle layer. K_V_7 channels were recently suggested to play a role in relaxation of erectile tissue and contribute to relaxations induced by the PDE5 inhibitor, sildenafil (Jepps et al., 2016). However, a selective blocker of K_V_7 channels, linopirdine failed to inhibit NS309 relaxation in the present study, and this agree with the previous observations of lack of effect of 10 μM NS309 on K_V_7.4 channels (Strøbæk et al., 2004). Moreover, a blocker of ATP-sensitive K^+^ channels, glybenclamide failed to change NS309-induced currents in HUVECs (Supplementary Figure S5B). However, in addition to opening K_Ca_2.3 and K_Ca_3.1 channels, 10–30 μM NS309 has been also reported to be a blocker of the voltage-dependent Ca^2+^ channels in bladder smooth muscle (Morimura et al., 2006) and voltage-dependent K^+^ (K_V_11.1) channels (Strøbæk et al., 2004). In the presence of high extracellular potassium and apamin, NS309 at 10 μM still induced 80% relaxations. Taken together these findings suggest that NS309 at low concentrations (0.5–1 μM) only leads to opening of K_Ca_2.x channels, while high concentrations of NS309 (>10 μM) relax rat CC by a K^+^ channel-insensitive mechanism which may involve blocking of voltage-dependent Ca^2+^ channels. Therefore, under the assumption of a volume of distribution on 2.0 L·kg^−1^ NS309 and NS4591 for the *in vivo* studies were administered in doses of 1 mg/kg to obtain plasma concentrations ~0.5–1 μM.

### Effect of openers of small and intermediate calcium-activated potassium channels on erectile function

Drugs currently used for treatment of erectile function, e.g., sildenafil, which inhibits PDE5 also causes relaxation in CC, and penile arteries involving activation of K_Ca_1.1 channels (Prieto, 2008). Also drugs, which enhance EDH type relaxation in human erectile tissue and restore erectile function in diabetic rats involve activation of K_Ca_2.3 and K_Ca_3.1 channels, although the exact mechanism of action of calcium dobesilate has to be clarified (Angulo et al., 2003b). NS11021 and NS8 are openers of K_Ca_1.1 channels and enhance erectile function in rats (Kun et al., 2009; González-Corrochano et al., 2013), but so far this is the first study reporting effect on erectile function of K_Ca_2.x and K_Ca_3.1 openers.

In previous studies we have validated the protocol applied for evaluation of drugs with effect on erectile function, and we found that sildenafil markedly enhanced the erectile responses (Kun et al., 2009). In the present study infusion of NS309 caused a small increase in intracavernous pressure, which is insignificant for erectile function and failed to change the increase in intracavernous pressure induced by electrical stimulation of the cavernous nerve. Although half-life has not been reported previously, both NS309 and NS4591 were found to have an effect on bladder activity by intravesical instillation (Pandita et al., 2006; Hougaard et al., 2009), but only NS4591 was examined and found to have effect on bladder activity by intraduodenal administration (Hougaard et al., 2009). Therefore, we examined whether NS4591 has a more pronounced effect on erectile function. Infusion of NS4591 markedly increased the response to submaximal stimulation of the cavernous nerve 3 min after infusion. The effect is comparable to the effect infusion of either the PDE5 inhibitor sildenafil or the K_Ca_1.1 channel opener, NS11021 (Kun et al., 2009). However, the duration of the effect of NS4591 as facilitator of erectile function seems limited, therefore further studies should be conducted to elucidate whether a drug, which is more selective for the K_Ca_2.3 channels, and with longer duration of action could provide a novel approach for treatment of erectile dysfunction.

Decrease in blood pressure and/or effect on cardiac K_Ca_2.3 and K_Ca_3.1 channels may limit the use of systemic administration of openers of these channels for erectile dysfunction. Infusion of other preferentially K_Ca_3.1 channel openers produce pronounced effects on blood pressure (Damkjaer et al., 2011). Although these studies were performed in anesthetized animals and it cannot be excluded that the outcome would be different in conscious animals, our results show that the effect on systemic blood pressure of NS309 was small, while the effect of NS4591 was marked compared to vehicle, but transient and disappeared within 60 s. Therefore, further studies will be required in hypertensive models to examine whether there can be an advantage of the blood pressure lowering effect of NS4591 and/or it possesses a risk in otherwise healthy patients with ED.

Based on studies in the transgenic K_Ca_2.3 overexpressing mice, it was suggested that K_Ca_2.3 channels play a critical role in the repolarization of the atria (Zhang et al., 2014), and the same was found in the isolated human atria (Skibsbye et al., 2014). Moreover, blocking K_Ca_2.3 channels could be of potential benefit in the treatment of supraventricular arrhythmias, e.g., atrial fibrillation as shown in rats and dogs (Diness et al., 2011; Qi et al., 2014). In addition to opening of K_Ca_2.x and K_Ca_3.1 channels, NS309 in cells patch also blocks human ether-a-go-go-related gene (hERG) potassium channels with Ki values of 1.3 μM (Strøbæk et al., 2004) while blocking of hERG channels was not observed for NS4591 (Hougaard et al., 2009). NS309 and NS4591 increased the amplitude of the P wave and shorten the P-R interval as well as the ventricular repolarization. These findings suggest that there is only a modest effect *in vivo* of these K_Ca_2.x and K_Ca_3.1 openers on the electrical activity of the heart. These results are not surprising as K_Ca_2.x channels have been suggested mainly to have a role as modulators in the repolarizing phase of the cardiomyocytes (Xu et al., 2003) and a modulation of the cardiomyocyte repolarizing phase would imply that the calcium events underlying activation of these channels, rather than drug-induced channel opening may play a major role on modulating the action potentials, and hence risk for development of atrial arrhythmia (Torrente et al., 2017). However, additional studies in other animal models, e.g., aged and/or ischemic heart models will be required to clarify this issue.

The present study suggests that openers of K_Ca_2.x and K_Ca_3.1 channels, e.g., NS4591 improves erectile function probably by enhancing the release of endothelium-dependent vasodilators, while the effects on mean arterial blood pressure, heart rate and cardiac electrical activity are modest. Currently, PDE5 inhibitors improve erectile function in patients with hypertension and erectile dysfunction, having less effect on erectile dysfunction in patients with diabetes (Francis and Corbin, 2011), and PDE5 inhibitors are contraindicated in patients taking nitrates in connection with ischaemic heart disease. Our study proposes a novel approach for treating erectile dysfunction and/or as an add-on to PDE5 inhibitor treatment in patients with diabetes.

## Ethics statement

Experiments with rats were conducted in accordance with the regulations of the Ethics Committee of the Animal Care and Use Committee at Aarhus University (Denmark) and to the Danish Animal Law (permission 2011/561-2011 and 2014-15-2934-01059), and also followed the ARRIVE guidelines.

## Author contributions

SC was involved in the design of the study and performed myograph experiments, immunoblotting, extraction of endothelial cells, and pharmacology experiments on anesthetized rats. He wrote a first draft of the manuscript and prepared the submission. IC performed isolation and culture of endothelial cells from CC, patch-clamp experiments and data analysis. She wrote the corresponding parts of the results and discussion. EH supervised immunoblotting and analysis of the results. She contributed to the writing of the manuscript. US was involved in the design and supervised the study and contributed to the writing of the manuscript. Everyone contributed with the final corrections on submission.

### Conflict of interest statement

The authors declare that the research was conducted in the absence of any commercial or financial relationships that could be construed as a potential conflict of interest. The reviewer PA and handling Editor declared their shared affiliation.

## Abbreviations

ED: Erectile dysfunction
PDE5: phosphodiesterase type 5
CC: *corpus cavernosum*
db/db leptin impaired type II diabetic mice: ZDF Zucker diabetic fatty rats
FMD: Flow mediated dilation
EDH: endothelium derived hyperpolarization
MAP: mean arterial pressure
NO: nitric oxide
ICP: intracavernous pressure
PICP: peak intracavernosal pressure
PDE5: phosphodiesterase type 5
HR: Heart rate
PSS: Physiological salt solution.
