# Supplementary material for: Small and Intermediate Calcium-Activated Potassium Channel Openers Improve Rat Endothelial and Erectile Function
Source: Front Pharmacol. 2017 Sep 20;8:660. doi: 10.3389/fphar.2017.00660 (PMC5619997; doi:10.3389/fphar.2017.00660)
Supplement: Supplementary file 1 [file Presentation1.PDF]

Small and Intermediate Calcium-Activated Potassium Channel Openers improve Rat Endothelial and Erectile Function

S Comerma-Steffensen[1,2*], I Carvacho[1,3,4], E R Hedegaard[1], and U Simonsen[1]

Supplementary figures

Figure S1A

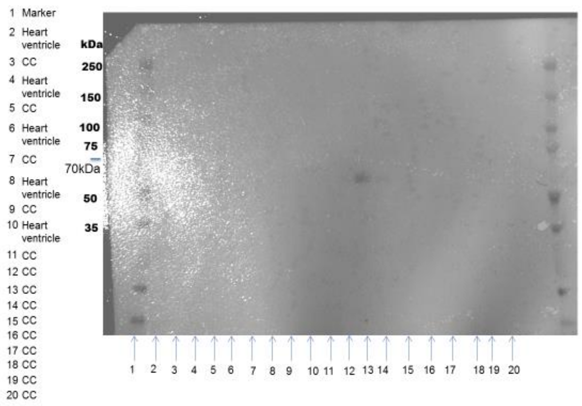

Figure S1B

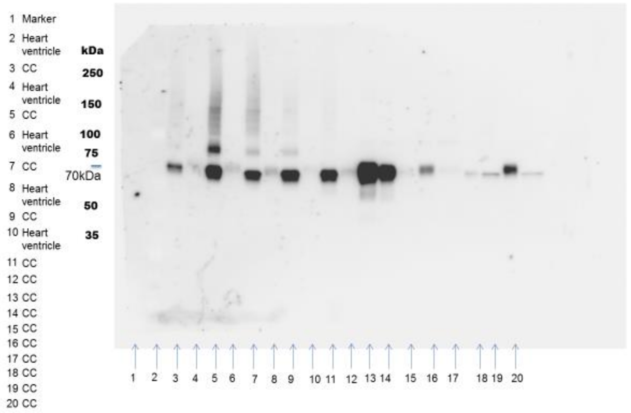

Figure S1C

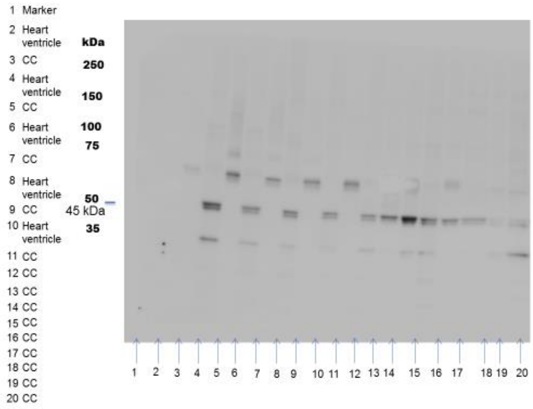

Suppl. Figure S1 A) Original membrane showing markers visualized by epi-luminescence, **B)** The same membrane exposed to cheminoluminescence to reveal the immunoblotting for $K_{Ca}2.3$ channels in the heart and *corpus cavernosum* of the rat and **C)** Original cheminoluminescense of

membrane of pan-actin antibody in heart and *corpus cavernosum* of the rat. Heart n=5 and *corpus cavernosum* n=14.

Figure S2A

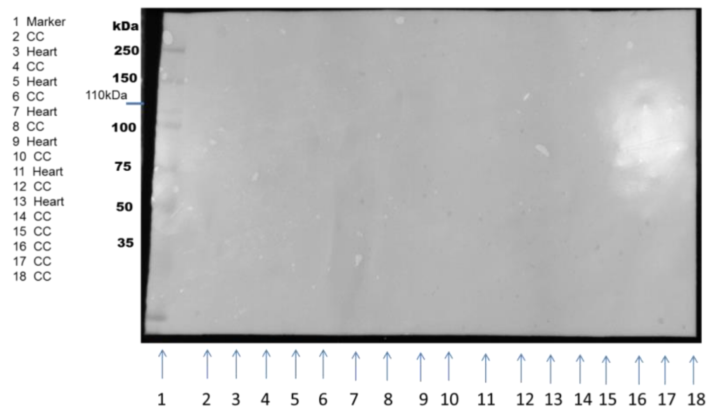

Figure S2B

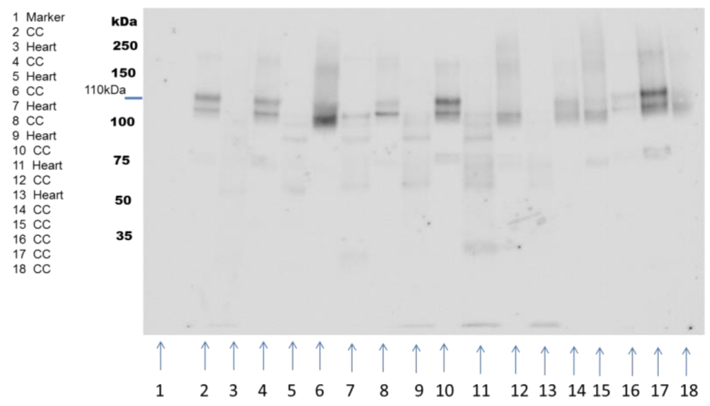

Figure S2C

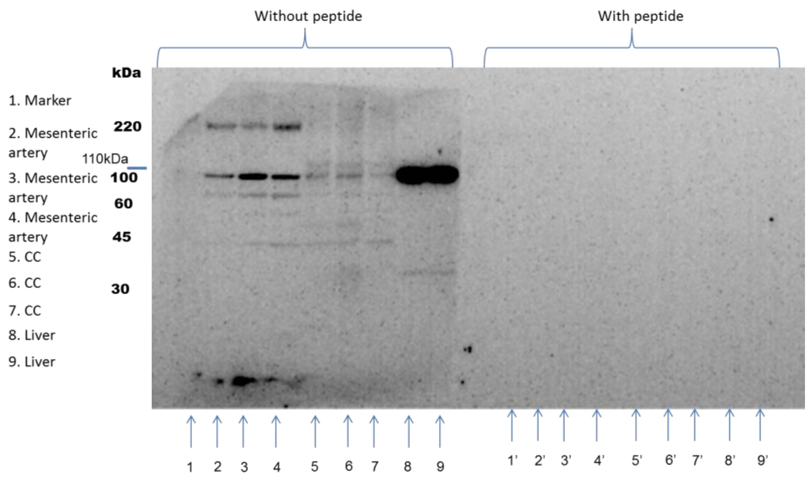

Suppl. Figure S2. A) Original membrane showing markers visualized by epi-luminescence, **B)** The same membrane exposed to chemiluminescence to reveal the immunoblotting for K_{Ca}1.1 samples in

heart and *corpus cavernosum* (CC) of the rat, **B)** Original chemiluminescence blotting of K_{Ca}1.1 antibody in heart and *corpus cavernosum* (CC) of the rat. Heart n=6 and *corpus cavernosum* n=11. C) Original chemiluminescence picture showing K_{Ca}1.1 alpha in *corpus cavernosum* (CC) of the rat. Mesenteric artery n=3, *corpus cavernosum* n=3 and Liver n=2. Left side membrane of is shown minus peptide and right side incubated with peptide used to raise the antibody for K_{Ca}1.1.

Figure S3A

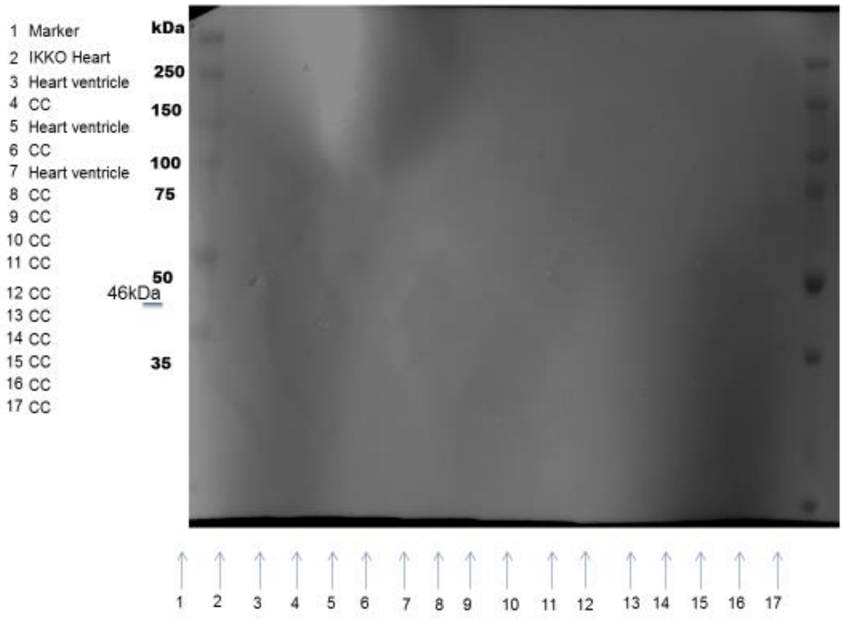

Figure S3B

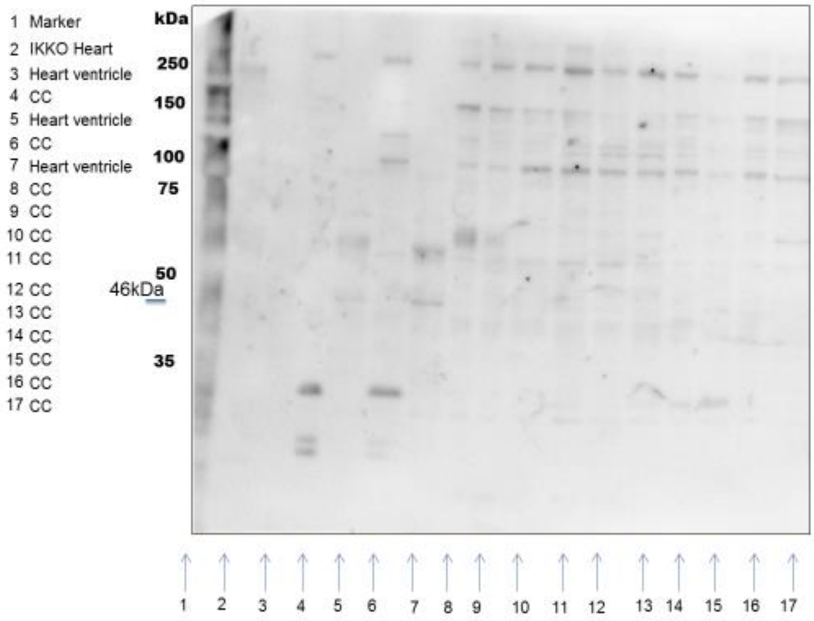

Suppl. Figure S3. A) Original membrane epi-illumination of K_{Ca}3.1 antibody in heart and *corpus cavernosum* (CC) of the rat **B)** Original chemiluminescence (blotting) of K_{Ca}3.1 antibody in heart and *corpus cavernosum* (CC) of the rat. K_{Ca}3.1 knockout mouse (IKKO) heart n=1, Rat heart n= 3 and *corpus cavernosum* n=12.

A B

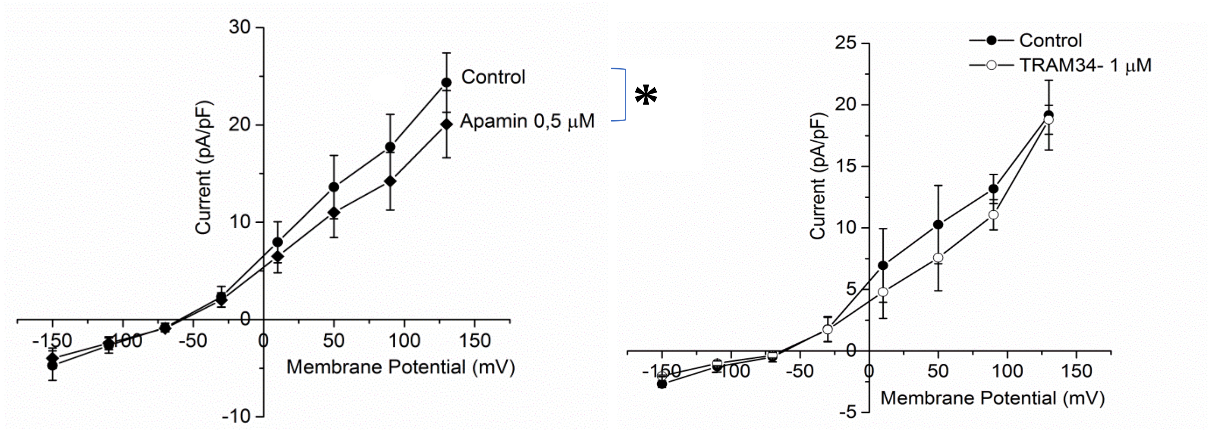

Suppl. Figure S4. Whole-cell voltage clamp recordings of currents evoked from voltage steps in primary cultures of endothelial cells from rat *corpus cavernosum*. (A) Whole-cell voltage clamp recordings of currents evoked from voltage steps (-140 to +140 mV) in control cells (*filled circles*) or in presence of 0,5 μM apamin (*filled diamonds*). (B) Whole-cell voltage clamp recordings of currents evoked from voltage steps (-140 to +140 mV) in control cells (*filled circles*) or in presence of 1 μM TRAM-34 (*open circles*). Cells were obtained from 3 different rats.

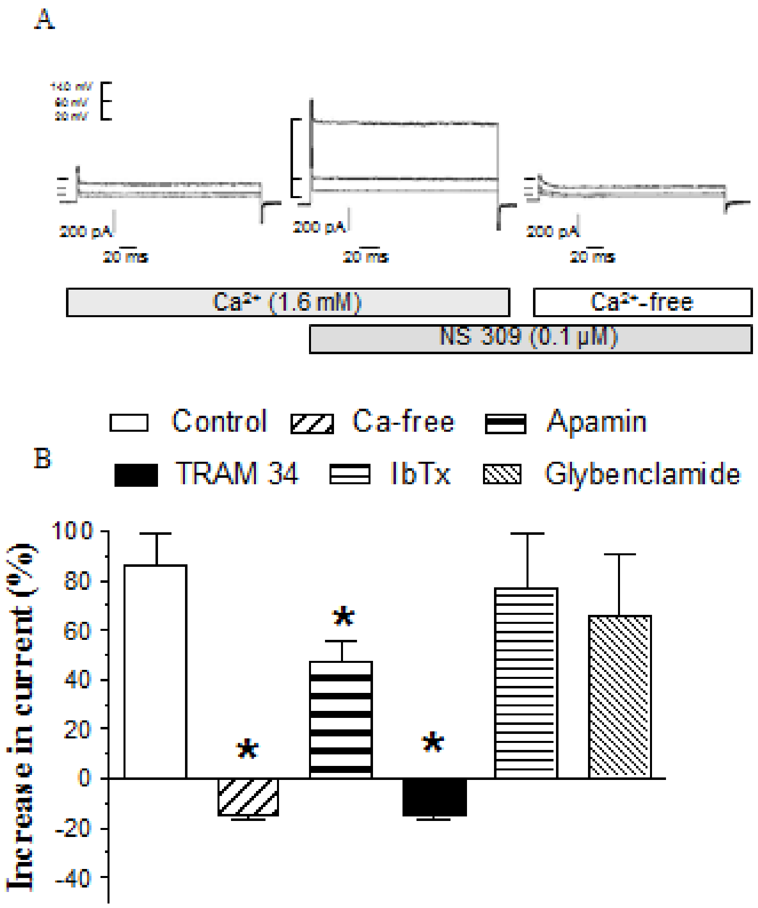

Suppl. Figure S5. (A) Whole cell patch clamp recording in human umbilical vein endothelial cell showing the effect of NS309 (0.1µM) in the presence (Ca^{2+}, 1.6 mM) and the absence of intracellular (Ca^{2+}-free) calcium. (B) Relative NS309 (0.1 µM) -induced increase in current in HUVECs in the presence and the absence (Ca-free) of calcium and the presence of calcium and different K channel blockers, apamin, TRAM-34 (1 µM), iberiotoxin (IbTx, 0.1 µM), and glybenclamide (0.1 µM). Modified from Stankevicius et al., 2011. Data are expressed as means ± S.E.M. *P≤0.05, significantly different from control. Human umbilical venous endothelial cells – HUVEC.

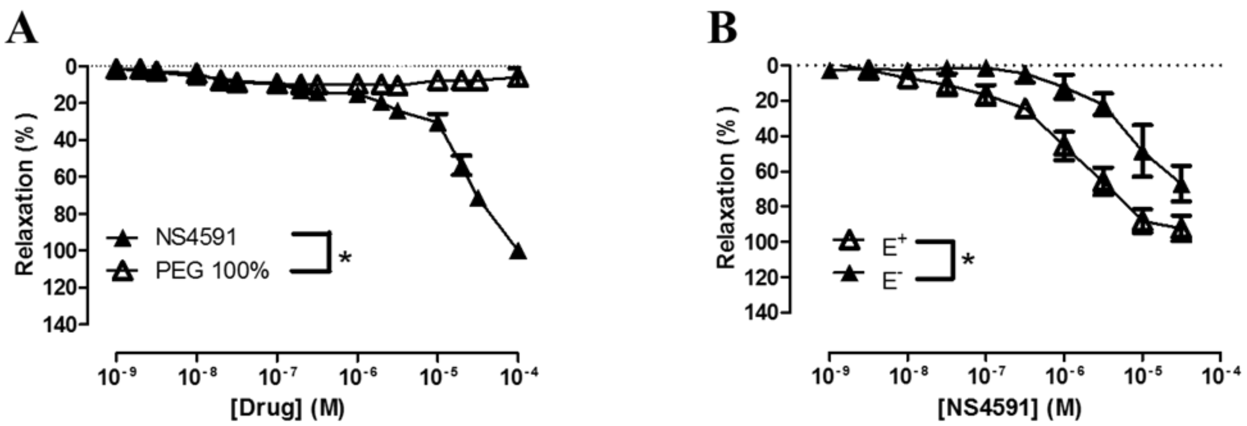

Suppl. Figure S6. Relaxations by induced by NS4591 in rat *corpus cavernosum*. (A) Effect of vehicle PEG 100% and of NS4591 in rat *corpus cavernosum* strips contracted with noradrenaline. (B) Relaxations induced by NS4591 in preparations with (n=5) and without endothelium (n=5). *P≤0.05, curves were significantly different, two-way ANOVA followed by a Bonferroni post-test.

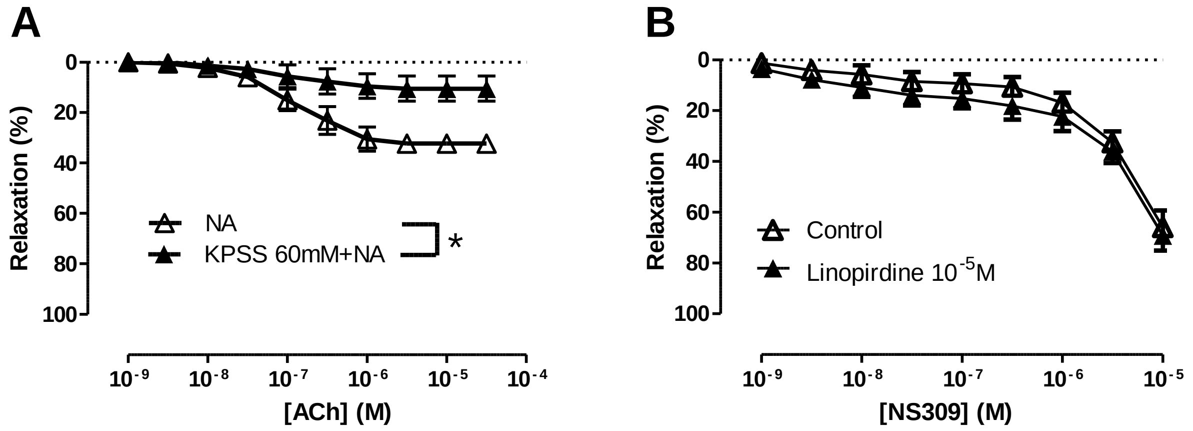

Suppl. Figure S7. Effect of high extracellular potassium on acetylcholine and NS309 relaxation of rat *corpus cavernosum.* Concentration-response curves for **A)** acetylcholine (ACh) in the absence (n=5) and the presence of high extracellular potassium (60 mM KPSS, n=5), **B)** NS309 in the absence (n=6) and the presence of a blocker of Kv7 channels. Linopirdine (10 µM, n=6). Data are expressed as means ± S.E.M. *P≤0.05, curves were significantly different, two-way ANOVA followed by a Bonferroni post-test.

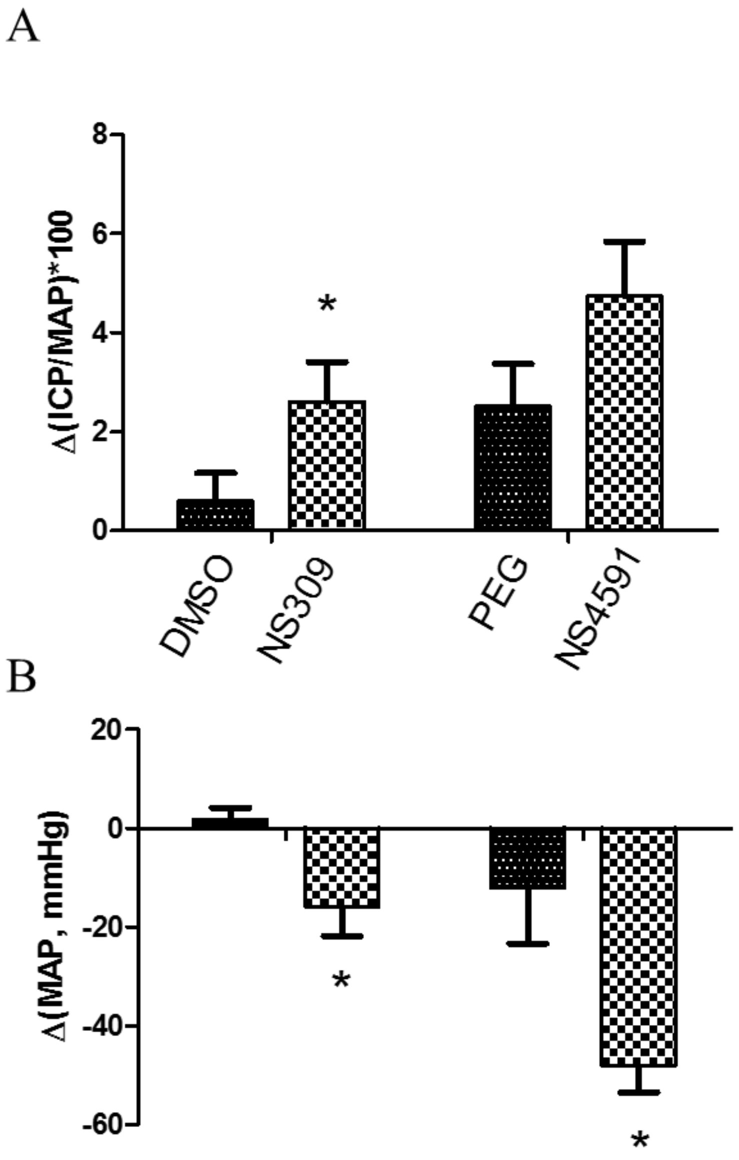

Suppl. Figure S8. Changes of and mean arterial pressure during and 1 min after infusion of NS309 and NS4591. (A) Changes in erectile function measured as intracavernosal pressure over mean arterial pressure (ΔICP/MAP*100) induced by (A) the vehicle DMSO (n=7), NS309 (1 mg/Kg, n=5), the vehicle PEG (n=7), and NS4591 (1 mg/kg, n=6), and (B) changes in mean arterial pressure (ΔMAP, mmHg) induced by DMSO, NS309 (1 mg/Kg), PEG and NS4591 (1 mg/kg). Data are expressed as means ± S.E.M. *P≤0.05, significantly different compared to vehicle using Student´s t-test.
